# Associations between accumulating job stressors, workplace social capital, and psychological distress on work-unit level: a cross-sectional study

**DOI:** 10.1186/s12889-023-16506-w

**Published:** 2023-08-16

**Authors:** Risto Nikunlaakso, Kaisa Reuna, Tuula Oksanen, Jaana Laitinen

**Affiliations:** 1 Finnish Institute of Occupational Health, 00250 Helsinki, Finland; 2 Finnish Institute of Occupational Health, 20520 Turku, Finland; 3 University of Eastern Finland, 70210 Kuopio, Finland; 4 Finnish Institute of Occupational Health, 90220 Oulu, Finland

**Keywords:** Work stress, Psychological distress, Social capital, Interaction, Work-units

## Abstract

**Background:**

Psychosocial job stressor studies usually examine one exposure at a time and focus on individual workers. In this study we examined the accumulation of work stressors in work units and its association with psychological distress (PD) on work-unit level. We also investigated whether high workplace social capital modifies the effect.

**Methods:**

We examined survey responses from 813 Finnish health and social services (HSS) work units, comprising 9 502 employees, in a cross-sectional study design. The survey was conducted in 2021. We calculated odds ratios for the association between accumulating job stressors and PD. We also analyzed the interaction between work stressors and the effect modification of high workplace social capital.

**Results:**

We found that HSS work units with high percentage of employees having high job demands and low rewards (OR 7.2, 95% CI 3.7, 13.8) have an increased risk of higher PD in the work unit. We also found indication of high social capital possibly modifying the effect of job stressors on PD. The results suggest that accumulated job stressors are associated with PD on work unit level, with excess risk for PD compared to the stressors acting separately.

**Conclusions:**

The results indicate that the effect of accumulating job stressors should be further studied on work-unit level. Participatory organizational-level and work-unit level interventions to tackle job stressors and to improve workplace social capital are warranted.

## Background

Health and social service (HSS) workers are in risk of burnout and other mental health problems due to workplace conditions [1–3]. The situation has worsened during the COVID-19 pandemic [4, 5]. Psychological distress (PD) is among the outcomes which are prevalent among HSS workers [6]. PD refers to symptoms of anxiety and depression, revealing a worrying situation which can still be intervened. In the workplace, psychosocial work stressors such as bullying [7], job strain [8], and accumulation of several work stressors [9] are found to increase risk for PD. Workers with high PD are in increased risk of sickness absence and early retirement [10–12].

Several reasons for focusing on the work-unit level in mental health promotion and work stress research exist. Most HSS workers perform their work duties in collaboration with other workers. Workplace conditions and the colleagues in work units are thus relevant for workers’ well-being and health [13]. Members of a work unit often perform similar tasks and thus share the demands of the work. For example, if a colleague in the same work unit is ill, job demands may increase for others. Members of a work unit also share many job resources: a good working atmosphere and workplace social capital, for example, can support workers in a demanding work situation [14]. Additionally, the negative consequences of high job demands and insufficient resources, for example burnout, can be emotionally contagious for the whole work unit [15, 16]. Analysis on the work unit level can also reduce the risk for reporting bias when using self-reported measures [17]. Focusing on exposures and outcomes the work unit level can produce important information on how work stress affects the work unit as a whole. As management in HSS organizations is based on line management and work units, the study of well-being at the work-unit level can provide important information to employers and the organization's management. This information is needed for managing mental well-being at work and preventing work disability. Using PD as a work-unit level outcome is also a novel perspective on mental health research.

A recent systematic review [18] found several multilevel studies which examine supraindividual (i.e., any work-unit level above an individual) job demands and job resources in shaping individual employee mental health. Studies which examine also organizational-level outcomes are much fewer. Moliner et al. [19] studied organizational justice and burnout in hotel work units, finding that on work unit level, interactional justice had a stronger relationship with burnout than procedural justice, which is opposite to the findings of studies using individual data. To our knowledge, however, no previous studies on mental health using work-unit level outcomes have been conducted among HSS workers.

Studies of employee mental health problems also usually study the effect of one psychosocial stressor at a time. Although interaction of psychosocial stressors has been studied in a few papers [20–23], the effect of accumulating job stressors on mental health is less often studied, despite a few recent papers [9, 24]. In the syndemics theory, introduced by Singer [25], accumulation of adverse events can create synergistic interaction: two or more epidemics interact synergistically, causing an excess burden to the population [26]. In the work environment, a syndemic effect can produce a vicious circle, i.e. a super-additive interaction of job stressors, which affects HSS workers’ mental health more than would be expected from the combination of coexisting job stressors [9]. In previous research, Juvani et al. [27] has shown that accumulation of job strain, effort-reward imbalance, and organizational injustice increase the risk for disability pensions both on individual and work-unit level. Additionally, Nikunlaakso et al. [9] showed that high job demands, low job rewards, and low workplace social capital synergistically interact and increase risk for PD on individual level. However, to our knowledge, no studies of synergistic interaction of job stressors on work-unit level, or studies of the moderating effect of workplace social capital on work-unit level exist.

Theoretically, job demands, rewards, and workplace social capital can be, depending on their level, either job stressors or job resources for workers. In the Job Demands-Resources (J-DR) model, the demanding aspects of work can lead to exhaustion from work, whereas job resources can help workers to cope with the negative influences of high demands [28]. Lack of resources, on the other hand, can complicate coping with job demands, which can lead to withdrawal behaviour [28]. Both the exhaustion process and withdrawal process can lead to deteriorated mental health [28]. In this paper, we defined both high demands of work (excessive workload and hurry) and a lack of job resources (low rewards) as job stressors; we also examined workplace social capital as a job resource helping workers cope with job stressors.

This study aimed, first, to examine the prevalence of work-unit level PD among HSS workers. Second, it aimed to investigate whether the accumulation of work-unit level high job demands and low rewards are associated with high prevalence of PD in work units. Third, it aimed to find out, whether high workplace social capital modifies the effect of high job demands and low rewards on PD. We studied the following hypotheses:

- H1. Accumulation of high work-unit level job demands and low work-unit level rewards increases the risk for work-unit level PD.
- H2. High workplace social capital modifies the effect between job stressors and PD, decreasing the risk for PD.

With these analyses we aim to provide evidence and guidance for workplace mental health interventions and future work unit-level studies.

## Methods

### Study population

This observational study analyses a total population sample of Finnish HSS employees, collected with a survey. The survey was undertaken 26.10.2021—28.11.2021 in six Finnish public health and social care organizations. 11 925 employees who were actively working in the organizations during that time responded to the survey. Workers on parental, sick or study leave were excluded from the eligible population. Total response rate of the survey was 62%. 90% of the respondents gave their con-sent to use the data for research. Total number of individual respondents was 10 914 and worked during the survey in 863 work units.

In the present study, we analyzed work units, which we identified using organizational structures of the participating organizations. After excluding work units with less than three employees (*N* = 50), the final data comprised 813 work units with 9 502 individual respondents.

The study was approved by the ethical committee of the Finnish Institute of Occupational Health. Participation in the survey was voluntary and consent to use the responses for scientific research was requested in the survey questionnaire.

### Variables

#### Outcome

The main outcome, psychological distress (PD), was measured using 4-item Patient Health Questionnaire (PHQ-4). PHQ-4 contains 2 items from the 9-item Patient Health Questionnaire (PHQ-9) and 7-item Generalized Anxiety Disorder (GAD-7) and is used as a screening instrument for psychological distress [29]. Responses were scored as 0 (“not at all”), 1 (“several days”), 2 (“more than half the days”), or 3 (“nearly every day”). The total score thus ranged from 0 to 12. The internal consistency of the scale was good: Cronbach’s alpha was 0.89. Following the rationale of Löwe et al. [30], individual survey responses with a PHQ-4 score of 6 or more were coded as psychologically distressed. We set work units with 20% or more cases as exposed to PD. The average PD level was 11%, rare enough outcome for interaction analyses [31].

#### Exposure variables

In the survey we used, job demands were measured by calculating a work unit mean from two items derived from the Job Content Questionnaire [32]: “An unreasonable amount of work is expected of me” and “I don’t have enough time to get my work done”. The response scale was five-level (1 = strongly agree to 5 = strongly disagree). The highest tertile of work units were set as exposed to high job demands and the remaining two tertiles as non-exposed. Cronbach’s alpha for the scale was good, 0.93. The average within-unit agreement index (rwg) was rather weak, 0.47, but the intra-class correlation ICC(1) intermediate, 0.18, meaning that 18% of total variance in job demands was between work units.

Job rewards were measured with three items from the effort-reward imbalance model [33]: “How much do you feel you get in return for work in terms of income and job benefits?”, “How much do you feel you get in return for work in terms of recognition and prestige?”, and “How much do you feel you get in return for work in terms of personal satisfaction?”. The response scale was five-level (1 = very much to 5 = not at all) and we calculated a mean of the scores for each work unit. The highest tertile of work units were set as exposed to low rewards and the remaining two tertiles as non-exposed. Cronbach’s alpha for the scale was rather good, 0.82. The average within-unit agreement index (rwg) was good, 0.76, and the intra-class correlation ICC(1) intermediate, 0.13.

Social capital was measured calculating a work unit mean from eight items: “We have a ‘we are together’ attitude”, “People feel understood and accepted by each other”, “We can trust our supervisor”, “People in the work unit cooperate in order to help develop and apply new ideas”, “Our supervisor treats us with kindness and consideration”, “Our supervisor shows concern for our rights as an employee”, “People keep each other informed about work-related issues in the work unit” and “Do members of the work unit build on each other's ideas in order to achieve the best possible outcome?” [34]. The response scale had five points (1 = strongly disagree to 5 = strongly agree in first seven items and 1 = to a very little extent to 5 = to a very great extent in last item). The highest tertile of work units were set as having high workplace social capital and the remaining two tertiles as having low workplace social capital. Cronbach’s alpha for the scale was good, 0.89. The average within-unit agreement index (rwg) was good, 0.80, and the intra-class correlation ICC(1) intermediate, 0.15.

#### Covariates

We used work unit mean age (< 45, 45–50, > 50; mean age was in most work units (89%) between 40 and 55 years), percentage of men in the work unit (0, 1–15, > 15; most work units were female-dominated), work unit size (< 20, ≥ 20), organizational field (nursing and health care, services for the elderly and the disabled, family and social services, and other services, including e.g. administration, cleaning, and support services and percentage of respondents with poor self-rated health (< 20, 20–49, ≥ 50) as covariates in the data. Age and sex of the individual workers were obtained from employee registers. Work unit size and organizational field were drawn from employee organizational structures. Perceived health was measured with a question: “how is your health”, with a 5-point scale: good, fairly good, average, fairly poor, and poor. Two latter points we combined for analysis.

### Statistical analysis

In this cross-sectional, observational study we used descriptive statistics and chi-square tests to identify differences in prevalence of psychological distress in work units and to analyze accumulation of job stressors in organizational fields. For main analysis we used stepwise logistic regression, which shows changes in associations after entering new variables in the model. In the first step we included only co-variates. In the second step, we added the accumulation of work stressors to analyze the effect of cumulative adversities, not interaction of the stressors [35]. In the third step, to analyze a possible buffering effect, we added high workplace social capital in the model.

We also analyzed interaction effect between high job demands and low rewards on PD. To analyze interaction effects both as a departure from multiplicativity and additivity, we calculated also relative excess risk due to interaction (RERI) effects [31]. We reported the results according to the recommendations of Knol and VanderWeele [36].

For analyses of PD prevalence, accumulation of job stressors and logistic regression analysis, we used the SPSS version 27.0.1.0. For calculations of RERI we used InteractionR package in R software.

## Results

The present study analyzed work-unit data collected with a survey of Finnish HSS employees. Psychological distress (PD) was most frequent (a) in work units with less than 20 employees and (b) in work units in the other fields (e.g. administration, cleaning, and support services, see Table 1). Additionally, work units with 50% or more employees having poor health, work units with mean age under 45, and work units with no male workers had slightly higher prevalence of PD.

**Table 1:** Work unit characteristics and the prevalence of psychological distress (PD)

**Characteristics**	**N**	**%**	**Prevalence of PD**	***P*-value**
All work units	813	100	11.1	
Mean age				0.502
< 45 years	314	38.6	12.4	
45‒50 years	331	40.7	10.9	
≥ 50 years	168	20.7	8.9	
Percentage of men				0.544
0	257	31.6	12.8	
1‒15	297	36.5	10.4	
≥ 15	259	31.9	10.0	
% with poor perceived health				0.119
< 20	341	41.9	12.6	
20‒49	386	47.5	8.8	
≥ 50	86	10.6	15.1	
Work unit size				< 0.001
< 20	427	52.5	16.2	
≥ 20	386	47.5	5.4	
Organisational field				0.027
Family and social	96	11.8	12.5	
Nursing and health care	257	31.6	8.6	
The elderly and the disabled	203	25.0	7.9	
Other	257	31.6	15.6	

Roughly half of work units were unexposed to any work stressor (Table 2). Roughly one sixth of work units were exposed to high job demands only, to low rewards only, or to accumulation of both stressors, respectively. Prevalence of high demands only was highest in family and social services, and the prevalence of low rewards only in services for the elderly and the disabled. The prevalence of both stressors was highest in nursing and health care services.

**Table 2:** Clustering of high demands and low rewards in work units in organisational fields of health and social services organisations

Stressors	High demands	Low rewards	Family and social	Nursing and health care	The elderly and the disabled	Other	HSS total
0	-	-	56.3%	47.1%	50.2%	53.3%	50.9%
1	+	-	19.8%	13.2%	14.3%	17.5%	15.6%
1	-	+	13.5%	18.3%	18.2%	13.6%	16.2%
2	+	+	10.4%	21.4%	17.2%	15.6%	17.2%

In an interaction analysis of high job demands and low rewards on PD (Table 3), we found that high job demands (OR 3.71, 95% CI 1.94, 7.10) were more strongly associated with PD than low rewards (OR 1.96, 95% CI 0.92, 4.16). The joint effect of high job demands and low rewards, however, had the strongest association with PD (OR 8.50, 95% CI 4.56, 15.84), which supports hypothesis H1. The RERI was 3.83 (95% CI − 0.65, 8.31), indicating a positive interaction: the estimated joint effect of high job demands and low rewards on the additive scale was greater than the sum of the estimated effects of high job demands and low rewards alone. However, the confidence interval shows that the interaction between high job demands and low rewards on PD on the additive scale is statistically insignificant. The measure of interaction on multiplicative scale, the ratio of ORs, was 0.69 (0.20, 2.40), indicating a weak but positive estimated joint effect on the OR scale of high job demands and low rewards, compared to the product of the estimated effects of high job demands and low rewards alone.

**Table 3:** Interaction between high job demands and low rewards on psychological distress

	Low rewards absent	Low rewards present	Effect of low rewards within the strata of high demands
	OR (95% CI)	OR (95% CI)	OR (95% CI)
High demands absent	1 (Reference)	1.96 (0.92, 4.16)	1.96 (0.92, 4.16)
High demands present	3.71 (1.94, 7.10)	8.50 (4.56, 15.84)	2.29 (1.19, 4.40)
Effect of high demands within the strata of low rewards	3.71 (1.94, 7.10)	4.34 (2.06, 9.14)	
Multiplicative scale	1.17 (0.43, 3.16)		
RERI	3.83 (-0.65, 8.31)		

*OR* Odds ratio, *CI* Confidence Interval, *RERI* Relative excess risk due to interaction

In stepwise logistic regression analysis of covariates, job stressors, and high social capital on PD (Table 4), the results of the first step show that work units with less than 20 employees are in higher risk for PD. The association becomes even stronger in steps two and three. Conversely, regarding the organisational field, healthcare work units are in lower risk for PD. Adding job stressors to the model in step 2 increases the explanatory power of the model. High job demands are strongly associated with PD both alone (OR 3.80, 95% CI 1.97, 7.35) and when accumulating with low rewards (OR 9.27, 95% CI 4.89, 17.57), as was expected from the interaction analysis presented in Table 3. High workplace social capital, when added to the model in step 3, seems to have a protective effect for PD (OR 0.31, 95% CI 0.17, 0.60). It also decreased the odds ratios of both job stressors and their accumulative effect. This indicates an effect modification of high social capital on job stressors and PD, as stated in hypothesis H2.

**Table 4:** Stepwise logistic regression analysis of associations between covariates, work stressors and high social capital, and psychological distress

	Step 1		Step 2		Step 3	
	OR	(95% CI)	OR	(95% CI)	OR	(95% CI)
Mean age (ref. = under 45)						
45–50	0.83	0.50, 1.37	0.79	0.46, 1.34	0.75	0.43, 1.28
Over 50	0.55	0.28, 1.06	0.56	0.28, 1.11	0.55	0.27, 1.12
Percentage of men (ref. = zero)						
1–15	1.14	0.64, 2.03	1.38	0.75, 2.53	1.39	0.75, 2.58
Over 15	0.86	0.48, 1.53	0.77	0.42, 1.41	0.80	0.43, 1.48
Work unit size (ref. = 20 or more)						
Less than 20	3.52**	2.06, 5.95	4.58**	2.58, 8.11	5.77**	3.20, 10.40
Organisational field (ref. = other)						
Family and social	0.68	0.34, 1.39	0.73	0.34, 1.53	0.67	0.31, 1.45
Nursing and health care	0.48*	0.27, 0.85	0.42*	0.23, 0.77	0.44*	0.24, 0.81
Elderly and disabled	0.55	0.29, 1.03	0.52	0.27, 1.01	0.53	0.27, 1.04
% with poor perceived health (ref. = under 20)						
20–49	0.61	0.36, 1.01	0.62	0.36, 1.07	0.61	0.35, 1.05
50 or more	1.07	0.53, 2.16	1.06	0.50, 2.22	1.12	0.52, 2.40
Job stressors (ref. = none)						
High demands only			3.80**	1.97, 7.35	3.43**	1.75, 6.70
Low rewards only			2.18*	1.02, 4.69	1.85	0.85, 4.01
High demands + low rewards			9.27**	4.89, 17.57	7.16**	3.72, 13.78
Job resources						
High Workplace Social Capital					0.31**	0.17, 0.60
Nagelkerke *R*^2^	0.10		0.22		0.25	

*OR* Odds ratio, *CI* Confidence Interval

^**^p < 0.001, *p < 0.05

## Discussion

### Main findings

Examining work units gives a novel viewpoint in job stressors and psychological distress among HSS employees. The present study has shown that the prevalence of PD is highest in work units with less than 20 employees, in work units in administration, cleaning, and support services, and especially in work units with the studied job stressors. Accumulation of high job demands and low rewards seems to increase the risk of PD on work unit level (OR 9.27, 95% CI 4.89, 17.57). Additionally, the results indicate an excess risk due to interaction of job stressors, producing a greater risk for PD compared to the stressors acting separately (RERI 3.83, 95% CI -0.65, 8.31). The interaction effect was, however, statistically insignificant, mainly due to limited data size. We also found that in work units with high workplace social capital the association between accumulating job stressors and PD was lower. When adding high workplace social capital in the model, the odds ratio for accumulation of high job demands and low rewards decreased from 9.27 (95% CI 4.89, 17.57) to 7.16 (95% CI 3.72, 13.78). The results indicate that high social capital may modify the negative effect of job stressors and protect work units for PD. The results thus support both hypothesis H1 and H2.

To our knowledge, this is the first study that has examined the synergistic interaction of job stressors and the moderating effect of social capital on work-unit level. Our findings of the excess risk of accumulating job stressors are in line with those of Juvani et al. [27], who found that clustering of work unit-aggregated job stressors—effort-reward imbalance, job strain and organizational injustice—increase the risk for disability retirement due to depressive disorders. Our finding of high workplace social capital as a preventive effect modifier between job stressors and PD is also mostly supported in previous studies. Low work unit-level social capital is associated with health impairment [37] and with higher risk of antidepressant purchases [13]. Furthermore, Moliner et al. [19] found that high interactional justice, which encompasses similar elements to supervisor’s role in the measure of workplace social capital, was associated with reduced work unit-level burnout symptoms. Kouvonen et al. [34] found, however, no association between aggregate-level social capital and depression and Török et al. [38] no evidence of work-unit level high social capital buffering the effect of high physical workload on long-term sickness absence. Generally, in most studies comparing individual and work unit-level effects, the work unit level effect has been smaller than the individual effect, which is contrary to the relatively strong associations found in this study.

According to Lazarus and Folkman [39] and Lazarus [40], stress is caused by the individual’s appraisal of a job demand, not the demand itself. A demand which is interpreted as an uncontrollable and aversive challenge causes individuals to react, provoking stress and making them anxious [41]. Based on the results of this study, we argue that a combination of high job demands and low rewards in the work unit can also affect individual perceptions of uncontrollable, aversive challenges. Furthermore, in the syndemics theory, adverse health conditions are hypothesized to co-occur in particular temporal or geographical contexts due to harmful social conditions [42]. Work units are a potential context where job stressors accumulate and interact synergistically, mutually enforcing the effect of each other and making the stressful environment even more challenging [9]. High work-unit-level workload can also increase the risk for workplace bullying, which further increases the risk for PD [43]. Contrastingly, in work units with high workplace social capital, supervisors and peers can give social support to cope with stressful situations and facilitate recovery from work during the working day.

### Limitations of the study and future research prospects

A few limitations or remarks should be considered when interpreting and generalizing the results of this study. First, as the within-unit agreement and intra-class correlations in PD were low, the results of this study must be interpreted with caution. A work unit with a few workers reporting PD have an increased mean PD even when other workers report no symptoms. We claim, however, that employers should observe these work units, because distress can lead to sickness absences [10] and thus increase workload for whole work unit. PD can also transmit to other workers [15, 16]. Second, we used a cross-sectional study design. This is sensible, as identifying work units in organizational structure over several study years is very challenging due to organizational changes. The causality of job stressors and PD in work units remains, however, unknown: work units with increased mean PD level can experience their work more demanding and less rewarding. Third, although the number of work units in our data was reasonable and sufficient for cross-tabulations and logistic regression analysis, it proved deficient for interaction analysis. The results of the interaction analysis had wide confidence intervals and produced a statistically insignificant result for RERI, and must therefore be interpreted as indicative. Fourth, although using work unit data reduces reporting bias, we were only able to use self-reported survey measures of exposures and outcome. On the other hand, PHQ-4 questionnaire is a valid instrument measuring PD [30]. Moreover, as PD represents symptoms of more severe mental health issues, it is a rational outcome measure in work unit-level, where workplace-level interventions can be used to mitigate PD. Fifth, we may not have included all essential covariates in our analysis. The analysis lacks, for example, organizational change, which is a potential confounder as it may affect both psychosocial work environment [44] and mental health [45, 46].

Our results and its limitations show that more studies with work-unit level outcome measures are needed, to gain more understanding of work unit-level job demands and job resources. Future studies should, for example, study and identify other job stressors that may accumulate on work unit-level and cause increased risk for mental health. Also other outcomes than PD should be studied. On the other hand, also the effect of other job resources than workplace social capital on work unit well-being should be studied.

### Theoretical and practical implications

The present study has shown that, first, psychosocial job stressors can be studied also on work-unit level. We found strong associations between stressors and PD. Second, we found that in addition to individual effect [9], high job demands and low job rewards interact synergistically also on work-unit level. Third, correspondingly to the findings of Nikunlaakso et al. [9], who found low social capital to increase risk for PD, high workplace social capital may protect stressed workers from PD.

This study offers also important practical implications. First, HSS employers should expand their focus in health and work ability promotion from individual coping to the functionality of work units. Many job stressors are jointly experienced and shared in work units, and the stressors could also be better tackled jointly in the same units. Second, it is vital to identify the most detrimental job stressors in HSS work units and to recognize the units and teams in which job stressors may accumulate. To accomplish this, HSS organizations should regularly measure well-being in their work units and the demands and resources in the units’ work environment. Third, equally important is to develop and implement work unit-level interventions which tackle stress factors and improve workplace social capital in work units. In addition to work-place interventions which focus on individual workers [47], co-creational, participatory interventions which both tackle job demands or strengthen resources and improve co-operation are urgently needed. An example of a co-creational intervention could be joint processing of staff survey results in the work unit and development of measures to tackle challenges in work processes and in the work environment.

## Conclusions

Mental health problems among HSS workers are in a rise, and effective interventions are needed. Most interventions are focused on individual workers, neglecting organizational-level interventions and interventions which tackle psychosocial job stressors. The novelty of this study is that the results provide hypotheses for the development of new co-creational interventions for HSS work units. The results indicate that accumulation of high job demands and low job rewards is detrimental for the mental well-being in work units, and thus should be a target in interventions. Furthermore, this study has shown that workplace social capital should be improved in the future interventions.

## Data Availability

The datasets analyzed in the current study are not publicly available due to legislative restrictions, as the data contains information that could compromise the privacy of the research participants. The data are, however, available from the corresponding author on reasonable request.

## Abbreviations

HSS: Health and social services
PD: Psychological distress
PHQ: Patient health questionnaire
RERI: Relative excess risk due to interaction
SoCa: Social capital
OR: Odds ratio
CI: Confidence interval
